# Use of Virtual Reality-Based Therapy in Patients with Urinary Incontinence: A Systematic Review with Meta-Analysis

**DOI:** 10.3390/ijerph19106155

**Published:** 2022-05-18

**Authors:** Anna Rutkowska, Silvia Salvalaggio, Sebastian Rutkowski, Andrea Turolla

**Affiliations:** 1 Department of Physical Education and Physiotherapy, Opole University of Technology, 45-758 Opole, Poland; a.rutkowska@po.edu.pl; 2 Laboratory of Rehabilitation Technologies, IRCCS San Camillo Hospital, 30126 Venice, Italy; silvia.salvalaggio@hsancamillo.it; 3 Padova Neuroscience Center, Università degli Studi di Padova, Via Orus 2/B, 35131 Padova, Italy; 4 Department of Biomedical and Neuromotor Sciences–DIBINEM, Alma Mater Studiorum Università di Bologna, Via Massarenti 9, 40138 Bologna, Italy; andrea.turolla@unibo.it; 5 Operative Unit Occupational Medicine, IRCCS Policlinico Sant’Orsola-Malpighi, Via Pelagio Palagi 9, 40138 Bologna, Italy

**Keywords:** virtual reality, urinary incontinence, pelvic floor muscle training, PFMT, quality of life, muscle function, UI symptoms

## Abstract

It is estimated that over 400 million people worldwide experience some form of urinary incontinence (UI). Pelvic floor muscle training (PFMT) is commonly used in cases of urine loss. Game therapy (GT) has been suggested as a new conservative modality for UI treatments. GT represents a form of virtual reality (VR) that allows users to interact with elements of a simulated scenario. The purpose of this review was to assess the potential of using VR-based PFMT in the treatment of UI with a particular focus on the impact of this form of therapy on the patients’ muscle function, symptoms of UI and quality of life (QoL). The following electronic databases were searched: PubMed, Embase, Cochrane Library, Scopus and Web of Science. Systematic review methods were based on the PRISMA (Preferred Reporting Items for Systematic Reviews and Meta-Analyses) statement. Electronic medical databases were searched from inception to 28 January 2021. From a total of 38 articles, 26 were analyzed after removing duplicates, then 22 records were excluded according to inclusion criteria and 4 were assessed as full texts. Finally, 2 randomized controlled trials (RCT) with 79 patients were included. For the International Consultation on Incontinence Questionnaire-Short Form (ICIQ-SF), the meta-analysis showed a significant difference in favor of the control condition (MD = 2.22; 95% CI 0.42, 4.01; I^2^ = 0%). Despite the popularity of the use of VR in rehabilitation, we found a scarcity of literature evaluating the application of VR in the field of UI therapy. Only one study matched all of the criteria established. The effects of VR training improved PFM function and QoL; however, these changes were comparable to those of traditional PFMT. It is not possible to reach final conclusions from one study; thus, further development of VR interventions in the field of UI treatments are needed.

## 1. Introduction

The number of individuals suffering from urinary incontinence (UI) is difficult to estimate. This problem affects women, men and children. To date, scientific reports estimate that 400 million people worldwide experience some form of incontinence [1,2,3].

Urinary incontinence is the complaint of involuntary leakage of urine. UI is classified into three types: Stress UI (SUI), Urge UI (UUI), and Mixed UI (MUI). As defined by the International Urogynecological Association (IUGA) and the International Continence Society (ICS), SUI may be defined as the involuntary leakage of urine in association with coughing, sneezing, or physical effort, i.e., during activities that increase abdominal pressure. SUI accounts for half of all UI, with most studies reporting 10% to 39% prevalence. Urge urinary incontinence (UUI) is a complaint of incontinence associated with a sudden need to urinate that is difficult to postpone (commonly referred to as “overactive bladder”). Isolated urgency urinary incontinence (UUI) is uncommon, with 1% to 7% prevalence. These two subtypes are so common that they often coexist as a combination of symptoms referred to as mixed urinary incontinence (MUI) [4,5]. According to data announced at the 6th International Consultation on Incontinence (ICI), UI-related problems in the general population range from 4% to 8%. The frequency of urinary incontinence increases with age: 24% to 45% of women report some level of incontinence, with prevalence improving from 7% to 37%, between the ages of 20 and 39 [6]. Moreover, daily incontinence is reported to range between 9% and 39% in women older than 60 years [6], with the stress UI peak of prevalence in the fifth decade. Increased risk of incontinence is associated with dysfunction of the bladder or pelvic floor muscles (PFM), pregnancy, childbirth, diabetes, menopause, gynecological surgeries and increased body mass index [3,6,7,8].

Incontinence is a large, often hidden problem among women. Unfortunately, the lack of early education, knowledge and awareness on the existence of this condition, and private advertising campaigns promoting passive management (e.g., the use of pads), lead to a misconception that UI constitutes a normal part of the aging process or is a consequence of pregnancy and childbirth. Thus, women significantly delay their first access to professionals or experts in the field for specialized medical and rehabilitation treatments [9]. UI is not only a serious disease, but also an unquestionable social problem that affects quality of life. UI limits social interaction and physical activity, and it is associated with impaired emotional and psychological wellbeing and sexual function [5,10,11].

Treatment of UI includes pharmaceutical interventions, surgery, incontinence devices, behavioral training and physiotherapy [12]. A key aspect of physiotherapy deals with pelvic floor muscle training (PFMT). One of the first people who described the impact of PFMT was the American gynecologist Arnold Kegel in the mid-20th century. Using pelvic floor muscle training with a biofeedback perinometer, Kegel has shown improved continence in his female patients [13]. In 2017, PFMT was defined by an International Standardization Committee as “an exercise to improve pelvic floor muscle endurance, strength, relaxation or a combination of these parameters” [14].

In healthy women, protection against urine loss due to activation of the pelvic floor muscles before or during exercise seems to be an automatic response that does not require conscious effort, but in the case of continence disorders, the correct mechanism of its functioning is deficient [15]. The researchers indicate that this mechanism can be recovered by PFMT [16]. Some studies show a positive correlation between PFMT and a reduction in incontinence episodes [5,10]. The literature suggests that short sessions (10–45 min) of PFMT with a frequency from 3 to 7 days per week may induce the greatest improvement in women with UI [17]. It has been shown that the appropriate analysis of PFM status prior to treatment, using non-invasive tests, could also improve outcomes [18]. Moreover, novel pre-operative PFMT programs enhanced post-surgical measures of pelvic floor muscle function, reduced PPI and improved QoL outcomes related to incontinence [19].

Prolonged training can be boring and discouraging for women, especially when they only work on one muscle group; thus, it seems interesting to include virtual reality in PFMT. The virtual environment provides an opportunity for guided rehabilitation using the attractiveness of the computer-generated world. Patients become more engaged and motivated to take part in the laborious and painstakingly long process leading to the recovery of their functions [20]. Jacobson suggests that there are four types of virtual reality: immersive virtual reality, desktop virtual reality (also called non-immersive), augmented virtual reality, and mixed virtual reality [21]. The concept of immersion means immersing the body and mind in a computer-simulated reality, which can dominate the real world. In order to make the user experience an exciting one, additional dedicated equipment can be used, such as: vibrating seats; smell emitters; deafening headphones and special goggles; and a so-called head-mounted display (HMD). VR is a technology that is constantly evolving and finding new applications in entertainment but also in medicine [22]. Some evidence supports the benefits of rehabilitation through various types of virtual reality in neurology, oncology, pulmonology and pediatrics [23,24,25,26]. Game therapy (GT) has been suggested as a new conservative modality for UI treatments. GT is a form of virtual reality used to create interactions between humans and machines that allows users to interact with elements of a simulated scenario [27,28].

The purpose of this review was to assess the effectiveness of virtual reality PFMT in the treatment of UI on patients’ quality of life (QoL), muscle function and symptoms. Systematic review methods were based on the PRISMA (Preferred Reporting Items for Systematic Reviews and Meta-Analyses) statement [29]. The protocol was registered a priori in the PROSPERO database under the following registration number: CRD42021229176.

## 2. Materials and Methods

### 2.1. Search Strategy

The following databases were searched: PubMed, Embase, Cochrane Library, Scopus and Web of Science. The search keywords and strategy used were: (“urinary incontinence” OR “stress urinary incontinence” OR “mixed urinary incontinence” OR “urgency urinary incontinence”) AND (“serious game” OR “video games” OR “virtual reality” OR “VR” OR “Virtual Reality” [mesh] OR “active video games” OR “head mounted display” OR “hmd” OR “virtual therapy” OR “virtual environment” OR “immersive” OR “non-immersive” OR “HTC” OR “Oculus” OR “Virtual Reality Exposure Therapy”) AND (“quality of life” OR “bladder diary” OR “perineometer” OR “vaginal dynamometer” OR “EMG” OR “electromyography” OR “ICIQ-OAB” OR “Qol” OR “maximal contraction” OR “Vaginal manometry” OR “ICIQ-UI SF” OR “International Consultation on Incontinence Questionnaire-UI” OR “Pad test”) for each database.

### 2.2. Study Selections

The primary criterion of the review was the use of virtual reality PFMT in the treatment of UI. The eligibility criteria were predefined by the authors (Table 1). Studies containing randomized controlled trials (RCTs), quasi RCT, non-RCT, Clinical Control Trials (CCT), pilot studies, and clinical control prospective studies were included. Nonetheless, case reports, reviews, study protocols, case series, clinical trials, and feasibility studies were not included in the analysis. The review included publications in English, Italian, Polish and Portuguese. Grey literature was also searched on Google Scholar databases, likewise in the reference lists of included articles. Eligibility criteria were defined using the PICO Framework [30].

- P—Population: adult individuals with urinary incontinence
- Intervention: virtual reality
- C—Comparison: no intervention or all other treatments
- Outcomes:
  ○ Assessment of muscle function (“maximal contraction”, “Vaginal manometry”, “vaginal dynamometer”, “perineometer”, “electromyography”, “EMG”)
  ○ Symptoms of UI (“urine loss”, “Pad test”, “bladder diary”)
  ○ Quality of life (“International Consultation on Incontinence Questionnaire-UI”, “ICIQ-UI SF”, “International Consultation on Incontinence Questionnaire Overactive Bladder”, “ICIQ-OAB”, “International Consultation on Incontinence Questionnaire Overactive Bladder Quality of Life Module”, “ICIQ-OABqol”).

### 2.3. Outcome Measures

Assessment of muscle function:

- Maximal voluntary contraction (MVC): The attempt to recruit as many fibers in a muscle as possible for the purpose of developing force. MVC of the pelvic floor can be assessed by vaginal palpation, dynamometers and manometers.
- Pelvic floor manometry/perineometer: Measurement of resting pressure or pressure rise generated during contraction of the PFM using a manometer connected to a sensor, which is inserted into the vagina, rectum or urethra. Pelvic floor manometric tools measure pressure in mmHg, hPa or cmH_2_O.
- Pelvic floor dynamometry: Measurement of PFM resting and contractile forces using strain gauges mounted on a speculum (a dynamometer), which is inserted into the vagina. Dynamometry measures force in Newton units (N = 1 kg × m/s^2^).
- EMG Electromyographic diagnosis: Made by evaluating the state of the muscle by recording and analyzing the electrical activity generated by the muscle. Surface electromyography: electrodes placed on the skin of the perineum or inside the urethra, vagina or rectum [14].
- Symptoms of UI (urine loss):
- Bladder diary: includes fluid intake, pad usage, number incontinence episodes, and the degree of incontinence.
- Pad testing: quantification of the amount of urine lost over the duration of testing by measuring the increase in weight of the perineal pads used (weighted pre- and post-testing) [14].
- Quality of life:
- The International Consultation on Incontinence Questionnaire Short Form (ICIQ-SF) is a short, new questionnaire (five questions) proposed by the World Health Organization with the aim of providing a clinically easy-to-use set of modules covering all aspects of the assessment of urinary incontinence severity and its impact on QoL. ICIQ-SF has a maximum score of 21; the higher the score, the more severe is the UI [31].
- The “International Consultation on Incontinence Questionnaire Overactive Bladder” (ICIQ-OAB) is a questionnaire for evaluating overactive bladder and related impact on quality of life (QoL) and outcome of treatment. It consists of 4 questions and an overall score ranging from 0 to 16, with greater values indicating increased symptom severity [32].
- The International Consultation on Incontinence Questionnaire Overactive Bladder Quality of Life Module (ICIQ-OABqol) explores in detail the impact of an overactive bladder on patients’ lives. It consists of 26 questions (overall score ranging from 25 to 160), with greater values indicating increased impact on quality of life [32].

### 2.4. Assessment of Risk of Bias in Included Studies

Two review authors independently assessed trials for eligibility using Rayyan software (https://www.rayyan.ai). A third review author resolved disagreements. The risk of bias for included studies was assessed using the ROB 2.0 tool from the Cochrane Collaboration [33], and the following five bias domains were considered: randomization, deviations from intended interventions, missing outcome data, measurement, and selection of reported results. Each domain was judged as low, some concern, or high risk based on responses to specific questions, resulting in an overall bias judgment for each specific study outcome assessed [33,34]. The data were transferred to an appropriate Excel spreadsheet, then presented graphically using the ROB 2.0 tool. The results of this evaluation were presented graphically using a device (Robvis) [35].

### 2.5. Data Extraction, Management and Synthesis

A data extraction form was filled with all the relevant data: author name, year of publication, participants (number and age), types of UI, treatment duration, treatment type, outcome measures, and main conclusions. RevMan 5.4.1 (The Cochrane Collaboration, London, UK) was used for the statistical analysis and meta-analysis. The Mean Difference (MD) outcome measures were used for analysis since all the selected studies used the ICIQ UI-SF tool. We conducted a meta-analysis based on a fixed model with a 95% confidence interval. Statistical heterogeneity was assessed with the I^2^ statistic, with a cut-off value at 50% and considering intervention and outcome measures.

## 3. Results

**Figure 1:**
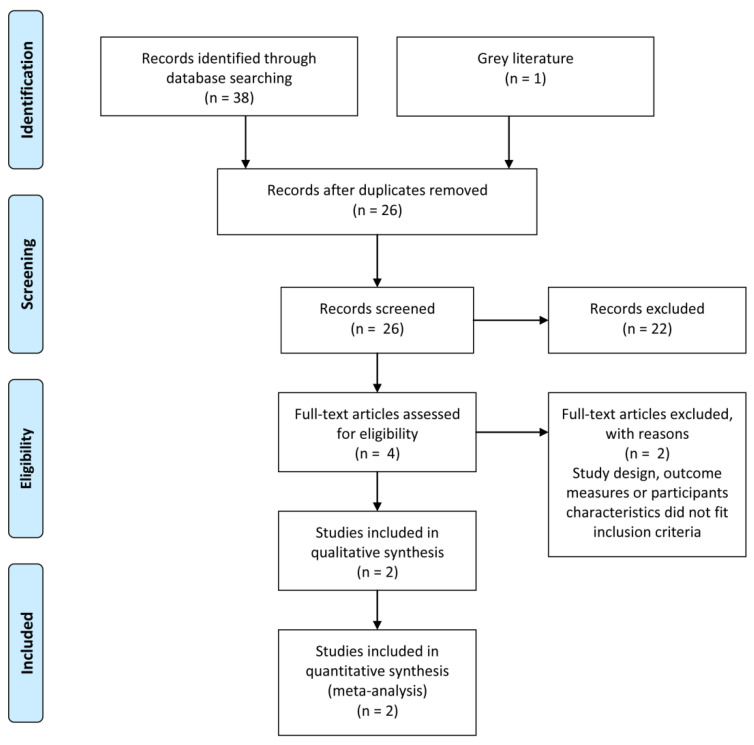
Flow diagram of the study.

Electronic medical databases were searched from inception to 28 January 2021. From a total of 39 articles, 26 were analyzed after removing duplicates. Then, 22 papers were excluded according to reported criteria; thus, 4 full texts were assessed. Finally, two more studies were excluded and two RCTs included [36]. The PRISMA flow diagram of the review process is displayed in Figure 1.

### 3.1. Included Studies

The Bezerra et al. study evaluated the effect of PFMT associated with game therapy on PFM pressure, urinary loss, and perception of improvement in women with mixed urinary incontinence (MUI). A single-blind randomized trial was conducted with 32 women, aged between 45 and 70 years, assigned to two groups: the PFMT group and the PFMT + GT group. Two sessions per week were performed for 8 weeks in each group. Each session lasted 40 min (PFM warmup for 5 min, then 35 min of strengthening exercise). The PFMT consisted of three modalities: breathing, abdominal and pelvic mobility exercises with PFM contraction. Interventions using GT + PFMT were performed by using the Wii Balance Board. The study used the Wii Fit Plus system and games: Lotus Focus and Penguin Slide, Basic Step and Hula Hoop. Data collections were performed before interventions and after the last treatment. The primary outcome was PFM pressure that was assessed by manometry. Secondary outcomes were a 1 h pad-test to quantify urinary losses through a pad, ICIQ-SF, which evaluates the frequency, severity, and impact of UI over the quality of life, and PGI-I that assesses a condition after the intervention. PFMT associated with GT did not show better improvements than PFMT in PFM pressure and urinary loss. Both interventions proved to be effective for the treatment of women with MUI. Treatments proposed in this study showed good acceptance, no withdrawal, easy applicability and were demonstrated to be effective [36].

A study by Martinho included two study groups in a randomized design. One group included postmenopausal women with MUI, while the other group included PFMT through kinesiotherapy. The authors used the following outcome measures to assess the effect of the intervention: electromyography, digital palpation, ICIQ UI-SF, ICIQ-OAB, and vaginal dynamometry. A significant increase in postmenopausal women’s muscle strength and endurance (digital palpation and vaginal dynamometry) and a concomitant decrease in their urinary symptoms (ICIQ UI-SF and ICIQ-OAB) were observed [37] (Table 2).

### 3.2. Excluded Studies

A study by Botelho et al. [38] was excluded from quantitative analysis. The authors included two study groups in a non-randomized study. The authors used different outcome measures to evaluate the intervention between groups. In fact, electromyography and digital palpation were used in the group of women without UI, while ICIQ UI-SF, ICIQ-OAB, vaginal dynamometry and digital palpation were used in the group of women with MUI. A feasibility study by Elliott et al., was excluded from quantitative analysis due to the lack of a control group [27].

### 3.3. Methodological Quality

**Figure 2:**
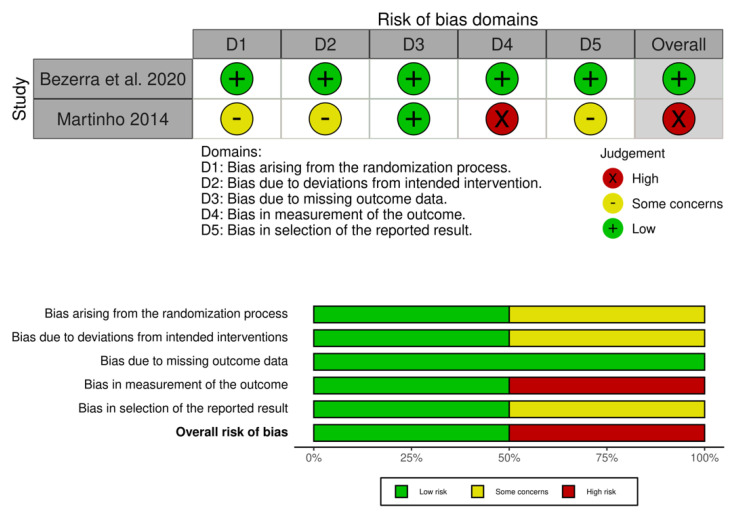
Risk of bias of included study.

Overall, the study by Bezerra et al. was considered as “low risk”, while the study by Martinho was considered as “high risk”. Detailed results of the risk of bias assessment are displayed in Figure 2.

### 3.4. Effects of Intervention

**Figure 3:**
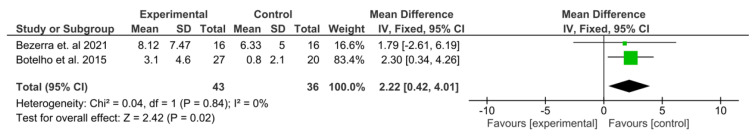
Comparison of experimental and control, according to ICIQ UI-SF. SD: standard deviation; 95% CI: 95% confidence interval.

Overall, the two studies enrolled 79 patients, and level, impact and perceived cause of symptoms of incontinence were analyzed. For the International Consultation on Incontinence Questionnaire-Short Form (ICIQ-SF), the meta-analysis showed a significant difference in favor of the control condition (MD = 2.22; 95% CI 0.42, 4.01; I^2^ = 0%) (Figure 3).

**Figure 4:**
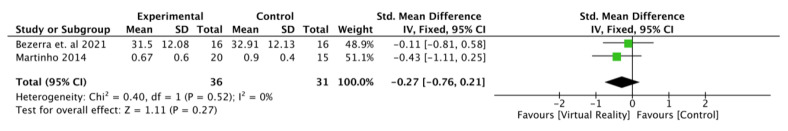
Comparison of experimental and control, according to dynamometry. SD: standard deviation; 95% CI: 95% confidence interval.

Both studies, with 67 patients overall, also included dynamometer measurements. In the included studies the dynamometry score was assessed using different units, and the analysis was performed using SMD with random model effect. No significant difference was found between VR treatment and standard procedures (SMD = −0.27; 95% CI −0.76, 0.21; I^2^ = 0%) (Figure 4). 

## 4. Discussion

This systematic review with meta-analysis aimed to assess the effectiveness of virtual reality PFMT in the treatment of UI in patients compared to standard PFMT. Despite the growing popularity of the use of virtual reality in rehabilitation, its use in clinical practice for PFMT in people with UI is still underdeveloped. A systematic review of the literature identified only two studies evaluating the effects of PFMT delivered with the support of virtual environments. Statistically significant benefits of standard PFMT were shown in the ICIQ-SF questionnaire, where treatment significantly decreased perceived impairments and symptoms. The results of this study showed the superiority of traditional training to therapy in a virtual environment. Yet, regarding the dynamometric test, despite the lack of statistical significance, the trend of increasing PFM strength was higher in the VR group. Further research is needed, exploring the use of modern technology with individuals suffering from various forms of UI. The results of risk of bias assessment revealed a diversity of conduct of the studies. It may be possible that the lower quality of Martinho’s study was due to the type of publication (a master’s thesis), whereas the original study by Bezerra et al. was prepared with a higher standard of scientific research papers, but this remains our speculation.

The basis of first-line UI treatment is PFMT [5]. Furthermore, there is an increased interest in the use of modern technologies in UI sufferers [39]. Due to poor knowledge of female anatomy and problems with proper contraction and relaxation of the pelvic floor muscles, biofeedback appears to be effective in therapy, which provides to the patient real-time feedback regarding its muscular function and contraction. Visual or sensory biofeedback are useful supports in training body districts with poor representation at the level of the central nervous system, such as the pelvis–perineal area; moreover, the effects on PFM function can be recorded. Biofeedback can allow faster and better results in pelvic floor therapy than traditional PFMT [40,41,42]. It is recommended that PFMT should last between 9 and 12 weeks [17,43].

Alternative technology solutions incorporating VR include mobile health (mHealth) applications. A number of smartphone apps available for iOS and Android devices now support PFM [44], while limited studies report on the use of mHealth systems in PFMT. Most likely, these involve simple systems based on the mobile app or apps connected with an endovaginal device. In a pilot study by Dufour et al., a 16-week PMFT was conducted to evaluate outcomes and determine aspects of acceptability and feasibility of an iBall device. The device assessed PFM strength and endurance. The authors reported no statistically significant differences between the experimental and control group on any outcomes [45]. Araujo et al. evaluated the adherence of home PFMT using a mobile application for women with UI. In this study, the authors did not use an endovaginal sensor, but only an application that displayed commands and images on a smartphone. The authors noted a higher adherence rate in the experimental group and improved UI outcomes [46]. Ong et al., in a randomized controlled pilot study, compared the Vibrance Kegel Device with a standard PFMT program, suggesting a superior benefit of this mHealth solution [47]. However, in all of the mentioned studies evaluating biofeedback-based training with a mobile app, the number of participants was small (23, 21, and 40, respectively).

An open question is whether the implementation of VR in the field of PFMT is clinically relevant. The number of papers concerning this subject is very limited. In the authors’ opinion, this is due to the lack of specialized therapeutic equipment dedicated to this kind of therapy. Concerning the example of neurorehabilitation, it can be noted that the development of software for VR therapy (game therapy or specialized) was dictated by the interest of clinicians in exploiting the advantages of technology-based augmented feedback for motor recovery and the recent development of technologies available in the market [48]. Nowadays, technology has advanced enough to provide a multi-sensory virtual reality experience. However, it is worth mentioning that costs associated with software development are not yet affordable, mainly due to the extended need of specialized human resources. Certain specialists (e.g., engineers, graphic designers, informaticians) are highly demanded by large companies [20]. In turn, those companies do not yet have business interests in developing applications for small groups of customers, as patients with UI may be perceived. Compared with traditional UI therapies, the advantages of VR therapy include the possibility of providing performance feedback and designing an individual setup that is easy to tailor and to quantify objective measures [23]. Moreover, the engagement and enjoyment of undertaking rehabilitation therapy is also an acknowledged advantage of using VR-based technologies. Thus, it can be assumed that the development of virtual rehabilitation in the UI area will develop in the coming years.

## 5. Conclusions

Despite the popularity of the use of virtual reality in rehabilitation, this literature review demonstrated large deficits in the field of UI therapy. In fact, only two studies matched all of the criteria established. The effects of training in VR were found to be inferior to traditional PFMT, which remains the standard care for UI treatment. To date, this field of research is still underdeveloped; nevertheless, the results of this review may guide the design of future randomized controlled studies investigating the effect of VR-based PFMT in patients suffering from UI.

## Figures and Tables

**Table 1:** Inclusion and exclusion criteria.

Inclusion Criteria	Exclusion Criteria
Study design: RCT, quasi RCT, CCT, non-RCT, pilot study, clinical control prospective study	Study design: case report, review, study protocol, case series, clinical trial, feasibility study
Population: adults and children with urinary incontinence, stress urinary incontinence, mixed urinary incontinence, urgency urinary incontinence.	Population: patients with schizophrenia, anorexia, autism, depression, cancers, anxiety, neurological disorders, healthy subjects without UI symptoms
Intervention: virtual reality immersive and non-immersive interventions	Intervention: robotics, smartphone applications
Comparison: all other treatments (non-VR) or no treatments	Lack of control group, equal intervention
Outcome: assessment of muscle function, Symptoms of UI, Quality of life	Outcome: all outcomes not related to urinary incontinence

**Table 2:** Characteristics of the included studies.

Reference	Participants/Age Range (yy)	Type of UI	VR Type	Treatments	Treatment Duration	Outcome Measures	Main Findings
Bezerra et al. 2021 [36]	32 women/45–75 yy	MUI	Wii Fit Plus	PFMT + GT (EG) (*n* = 16) PFMT (CG) (*n* = 16)	8 weeks	Manometry, QoL-ICIQ-SF, 1 h pad-test	PFMT associated with GT did not show better improvements than PFMT isolated in PFM quality of life, pressure and urinary loss. Both interventions proved to be effective for the treatment of women with MUI.
Martinho 2014 [37]	47 women/53–69 yy	MUI	Wii Fit Plus	PFMT + VR (*n* = 27) PFMT (*n* = 20)	5 weeks	ICIQ UI-SF, ICIQ-OAB, Dynamometry, DP	PFMT through virtual reality equates to PFMT through kinesiotherapy regarding the improvement of pelvic floor muscle strength, voiding symptoms, anterior wall prolapse and quality of life, proving to be effective for postmenopausal women.

Abbreviations: CG, control group; DP, digital palpation; EG, experimental group; GT, game therapy; ICIQ-OAB, International Consultation on Incontinence Questionnaire–Overactive Bladder; ICIQ-SF, International Consultation on Incontinence Questionnaire-Short Form; MUI, mixed urinary incontinence; PFMT, pelvic floor muscle training; PFM, pelvic floor muscle; QoL, quality of Life; yy, years.

## Data Availability

Not applicable.

## References

[1] Abrams P., Andersson K.E., Apostolidis A., Birder L., Bliss D., Brubaker L., Cardozo L., Castro-Diaz D., O’Connell P.R., Cottenden A. (2018). 6th International Consultation on Incontinence. Recommendations of the International Scientific Committee: Evaluation and Treatment of Urinary Incontinence, Pelvic Organ Prolapse and Faecal Incontinence. Neurourol. Urodyn..

[2] Griebling T.L. (2011). Worldwide Prevalence Estimates of Lower Urinary Tract Symptoms, Overactive Bladder, Urinary Incontinence, and Bladder Outlet Obstruction. Bju Int..

[3] Buckley B.S., Lapitan M.C.M. (2010). Prevalence of Urinary Incontinence in Men, Women, and Children-Current Evidence: Findings of the Fourth International Consultation on Incontinence REPLY. Urology.

[4] Haylen B.T., de Ridder D., Freeman R.M., Swift S.E., Berghmans B., Lee J., Monga A., Petri E., Rizk D.E., Sand P.K. (2010). An International Urogynecological Association (IUGA)/International Continence Society (ICS) Joint Report on the Terminology for Female Pelvic Floor Dysfunction. Neurourol. Urodyn..

[5] Dumoulin C., Cacciari L.P., Hay-Smith E.J.C. (2018). Pelvic floor muscle training versus no treatment, or inactive control treatments, for urinary incontinence in women. Cochrane Database Syst. Rev..

[6] Tran L.T., Puckett Y. (2020). Urinary Incontinence.

[7] Stewart W.F., Hirsh A.G., Kirchner H.L., Clarke D.N., Litchtenfeld M.J., Minassian V.A. (2014). Urinary Incontinence Incidence: Quantitative Meta-Analysis of Factors that Explain Variation. J. Urol..

[8] Wu J.M., Hundley A.F., Fulton R.G., Myers E.R. (2009). Forecasting the prevalence of pelvic floor disorders in U.S. Women: 2010 to 2050. Obs. Gynecol..

[9] Siddiqui N.Y., Levin P.J., Phadtare A., Pietrobon R., Ammarell N. (2014). Perceptions about female urinary incontinence: A systematic review. Int. Urogynecol. J..

[10] Radziminska A., Straczynska A., Weber-Rajek M., Styczynska H., Strojek K., Piekorz Z. (2018). The impact of pelvic floor muscle training on the quality of life of women with urinary incontinence: A systematic literature review. Clin. Interv. Aging.

[11] Preda A., Moreira S. (2019). Stress Urinary Incontinence and Female Sexual Dysfunction: The Role of Pelvic Floor Rehabilitation. Acta Med. Port..

[12] Ostle Z. (2016). Assessment, diagnosis and treatment of urinary incontinence in women. Br. J. Nurs..

[13] Kegel A.H. (1948). Progressive resistance exercise in the functional restoration of the perineal muscles. Am. J. Obs. Gynecol..

[14] Bo K., Frawley H.C., Haylen B.T., Abramov Y., Almeida F.G., Berghmans B., Bortolini M., Dumoulin C., Gomes M., McClurg D. (2017). An International Urogynecological Association (IUGA)/International Continence Society (ICS) joint report on the terminology for the conservative and nonpharmacological management of female pelvic floor dysfunction. Int. Urogynecol. J..

[15] Peschers U.M., Vodusek D.B., Fanger G., Schaer G.N., DeLancey J.O.L., Schuessler B. (2001). Pelvic muscle activity in nulliparous volunteers. Neurourol. Urodyn..

[16] Hayes W. (2008). Evidence-Based Physical Therapy for the Pelvic Floor-Bridging Science and Clinical Practice.

[17] Garcia-Sanchez E., Avila-Gandia V., Lopez-Roman J., Martinez-Rodriguez A., Rubio-Arias J.A. (2019). What Pelvic Floor Muscle Training Load is Optimal in Minimizing Urine Loss in Women with Stress Urinary Incontinence? A Systematic Review and Meta-Analysis. Int. J. Environ. Res. Public Health.

[18] Boni A., Cochetti G., Del Zingaro M., Paladini A., Turco M., Rossi de Vermandois J.A., Mearini E. (2019). Uroflow stop test with electromyography: A novel index of urinary continence recovery after RARP. Int. Urol. Nephrol..

[19] Milios J.E., Ackland T.R., Green D.J. (2019). Pelvic floor muscle training in radical prostatectomy: A randomized controlled trial of the impacts on pelvic floor muscle function and urinary incontinence. BMC Urol..

[20] Rutkowska A., Rutkowski S., Szczepanska-Gieracha J. (2020). The use of total immersion in the rehabilitation process. Med. Rehabil..

[21] Jacobson L., Swadley R. (1993). Welcome to the virtual world. On the Cutting Edge of Technology.

[22] Kristiansen L., Magnussen L.H., Wilhelmsen K.T., Maeland S., Nordahl S.H.G., Clendaniel R., Hovland A., Juul-Kristensen B. (2019). Efficacy of intergrating vestibular rehabilitation and cognitive behaviour therapy in persons with persistent dizziness in primary care- a study protocol for a randomised controlled trial. Trials.

[23] Rutkowski S., Kiper P., Cacciante L., Cieslik B., Mazurek J., Turolla A., Szczepanska-Gieracha J. (2020). Use of virtual reality-based training in different fields of rehabilitation: A systematic review and meta-analysis. J. Rehabil. Med..

[24] Rutkowski S., Rutkowska A., Jastrzebski D., Racheniuk H., Pawelczyk W., Szczegielniak J. (2019). Effect of Virtual Reality-Based Rehabilitation on Physical Fitness in Patients with Chronic Obstructive Pulmonary Disease. J. Hum. Kinet..

[25] Bonnechere B., Jansen B., Omelina L., Van Sint Jan S. (2016). The use of commercial video games in rehabilitation: A systematic review. Int. J. Rehabil. Res..

[26] Jastrzebski D., Zebrowska A., Rutkowski S., Rutkowska A., Warzecha J., Ziaja B., Palka A., Czyzewska B., Czyzewski D., Ziora D. (2018). Pulmonary Rehabilitation with a Stabilometric Platform after Thoracic Surgery: A Preliminary Report. J. Hum. Kinet..

[27] Elliott V., de Bruin E.D., Dumoulin C. (2015). Virtual Reality Rehabilitation as a Treatment Approach for Older Women With Mixed Urinary Incontinence: A Feasibility Study. Neurourol. Urodyn..

[28] Martinho N.M., Silva V.R., Marques J., Carvalho L.C., Iunes D.H., Botelho S. (2016). The effects of training by virtual reality or gym ball on pelvic floor muscle strength in postmenopausal women: A randomized controlled trial. Braz. J. Phys..

[29] Liberati A., Altman D.G., Tetzlaff J., Mulrow C., Gotzsche P.C., Ioannidis J.P.A., Clarke M., Devereaux P.J., Kleijnen J., Moher D. (2009). The PRISMA statement for reporting systematic reviews and meta-analyses of studies that evaluate healthcare interventions: Explanation and elaboration. BMJ Br. Med. J..

[30] Methley A.M., Campbell S., Chew-Graham C., McNally R., Cheraghi-Sohi S. (2014). PICO, PICOS and SPIDER: A comparison study of specificity and sensitivity in three search tools for qualitative systematic reviews. BMC Health Serv. Res..

[31] Hajebrahimi S., Corcos J., Lemieux M.C. (2004). International consultation on incontinence questionnaire short form: Comparison of physician versus patient completion and immediate and delayed self-administration. Urology.

[32] Donovan J., Abrams P., Peters T., Kay H., Reynard J., Chapple C., de la Rosette J., Kondo A. (1996). The ICS-‘BPH’ study: The psychometric validity and reliability of the ICSmale questionnaire. BJU.

[33] Sterne J.A.C., Savovic J., Page M.J., Elbers R.G., Blencowe N.S., Boutron I., Cates C.J., Cheng H.Y., Corbett M.S., Eldridge S.M. (2019). RoB 2: A revised tool for assessing risk of bias in randomised trials. BMJ.

[34] Higgins J.P., Altman D.G., Gotzsche P.C., Juni P., Moher D., Oxman A.D., Savovic J., Schulz K.F., Weeks L., Sterne J.A. (2011). The Cochrane Collaboration’s tool for assessing risk of bias in randomised trials. BMJ.

[35] McGuinness L.A., Higgins J.P.T. (2021). Risk-of-bias VISualization (robvis): An R package and Shiny web app for visualizing risk-of-bias assessments. Res. Synth. Methods.

[36] Bezerra L.O., de Oliveira M.C.E., da Silva Filho E.M., Vicente da Silva H.K., Menezes de Oliveira G.F., da Silveira Goncalves A.K., Pegado R., Micussi M. (2021). Impact of Pelvic Floor Muscle Training Isolated and Associated with Game Therapy on Mixed Urinary Incontinence: A Randomized Controlled Trial. Games Health J..

[37] Martinho N. (2014). O Treinamento Por Meio De Realidade Virtual Melhora A Funcionalidade Dos Músculos Do Assoalho Pélvico De Mulheres Na Pós-Menopausa?. https://bdtd.unifal-mg.edu.br:8443/bitstream/tede/588/5/Disserta%C3%A7%C3%A3o%20de%20Natalia%20Miguel%20Martinho.pdf.

[38] Botelho S., Martinho N.M., Silva V.R., Marques J., Carvalho L.C., Riccetto C. (2015). Virtual reality: A proposal for pelvic floor muscle training. Int. Urogynecol. J..

[39] Oliveira M.C.E., Bezerra L.O., Melo Angelo P.H., de Oliveira M.C., Silva-Filho E., Ribeiro T.S., Pegado R., Micussi M. (2020). Game therapy a new approach to treat women facing mixed urinary incontinence: A study protocol. Neurourol. Urodyn..

[40] Oliveira M., Ferreira M., Azevedo M.J., Firmino-Machado J., Santos P.C. (2017). Pelvic floor muscle training protocol for stress urinary incontinence in women: A systematic review. Rev. Assoc. Med. Bras..

[41] Aukee P., Immonen P., Penttinen J., Laippala P., Airaksinen O. (2002). Increase in pelvic floor muscle activity after 12 weeks’ training: A randomized prospective pilot study. Urology.

[42] Glavind K., Nohr S.B., Walter S. (1996). Biofeedback and physiotherapy versus physiotherapy alone in the treatment of genuine stress urinary incontinence. Int. Urogynecol. J. Pelvic Floor Dysfunct..

[43] (2019). Urinary Incontinence and Pelvic Organ Prolapse in Women: Management.

[44] Dantas L.O., Carvalho C., Santos B.L.J., Ferreira C.H.J., Bo K., Driusso P. (2021). Mobile health technologies for the management of urinary incontinence: A systematic review of online stores in Brazil. Braz. J. Phys..

[45] Dufour S., Fedorkow D., Kun J., Deng S.X., Fang Q. (2019). Exploring the Impact of a Mobile Health Solution for Postpartum Pelvic Floor Muscle Training: Pilot Randomized Controlled Feasibility Study. JMIR Mhealth Uhealth.

[46] Araujo C.C., Marques A.A., Juliato C.R.T. (2020). The Adherence of Home Pelvic Floor Muscles Training Using a Mobile Device Application for Women with Urinary Incontinence: A Randomized Controlled Trial. Female Pelvic Med. Reconstr. Surg..

[47] Ong T.A., Khong S.Y., Ng K.L., Ting J.R., Kamal N., Yeoh W.S., Yap N.Y., Razack A.H. (2015). Using the Vibrance Kegel Device With Pelvic Floor Muscle Exercise for Stress Urinary Incontinence: A Randomized Controlled Pilot Study. Urology.

[48] Szczepanska-Gieracha J., Cieslik B., Rutkowski S., Kiper P., Turolla A. (2020). What can virtual reality offer to stroke patients? A narrative review of the literature. NeuroRehabilitation.

